# Impact of an educational intervention on improving maternity nurses’ knowledge and attitudes toward postpartum depression: a quasi-experimental study

**DOI:** 10.25122/jml-2024-0147

**Published:** 2024-08

**Authors:** Amal Ibrahim Khalil, Jana Omar Saad, Raghad Alghamdi, Nagham Hamza Bahatheq, Shorouq Aied Alhrthy

**Affiliations:** 1 Psychiatric and Mental Health Nursing Department, College of Nursing, King Saud Bin Abdulaziz University for Health Sciences, Jeddah, Saudi Arabia,; 2 King Abdullah International Medical Research Center, Jeddah, Saudi Arabia; 3 Faculty of Nursing, Menoufia University, Shebin El Kom, Egypt; 4 College of Nursing, King Saud Bin Abdulaziz University for Health Sciences, Jeddah, Saudi Arabia

**Keywords:** Postpartum depression, educational intervention, maternity nurses, knowledge and attitudes, quasi-experimental design, PPD, Postpartum Depression, PODLIS, Postpartum Depression Literacy Scale, CONJ, College of Nursing, Jeddah, KAIMRC, King Abdulaziz Medical Research Center, IRB, International Research Board, SN1, Staff Nurse 1, SN2, Staff Nurse 2, NGHA, National Guard Health Affairs

## Abstract

Maternal mental health is a serious issue that affects both mothers and infants, necessitating increased knowledge and awareness among healthcare providers. This study assessed the effect of an educational intervention on maternity nurses' knowledge and attitudes towards postpartum depression (PPD) using a quasi-experimental pre/post-one-group design. The sample consisted of 120 maternity nurses recruited conveniently from different maternity departments at the Ministry of National Guard hospital. The study used two valid and reliable instruments: The Postpartum Depression Literacy Scale (PODLIS) by Mirsalimi *et al*. (2020) and the Attitudes Scale adapted from Kang *et al*. (2019). The participants, predominantly aged 30-39 with at least a bachelor's degree and holding SN1 nurse status, were mainly from Malaysia. The intervention significantly increased PPD knowledge in all domains, with the greatest improvement in risk factors and causes (10.09%). Positive associations existed between the pre-intervention attitude scale and professional help, recognition facilitation, and overall PPD knowledge (*P* < 0.05). Post-intervention, attitudes correlated positively with understanding risk factors and causes and facilitating recognition (*P* < 0.05). There were significant variations in the change of overall PPD knowledge based on the participants’ nationality (*P* < 0.05), and attending a PPD workshop significantly affected the change of attitude (*P* < 0.05). The study concluded the beneficial effects of the educational intervention on both knowledge and attitudes regarding PPD among maternity nurses. Customized educational programs are essential for preparing healthcare professionals with the necessary competencies and comprehension to deal with PPD effectively.

## INTRODUCTION

Pregnancy and childbirth mark substantial periods in a mother’s life, as they encompass not only hormonal, physical, and social but also biological and emotional changes. Combined with sociocultural factors, such periods can often be linked to the onset of postpartum depression (PPD) [1].

According to the Diagnostic and Statistical Manual of Mental Disorders (DSM–5), postpartum depression is characterized by a major depressive episode that occurs either during pregnancy or within four weeks of delivery. The diagnosis is made when the patient exhibits at least five symptoms for 2 weeks [2].

The etiology of postpartum depression is complex and involves various factors, including psychological, obstetric, social, and lifestyle elements. This condition can affect any woman with a history of anxiety, depression, or obstetric complications such as emergency cesarean section or preterm labor. Additionally, lack of social support, experiences of domestic violence, and inadequate eating and sleeping habits can all contribute to the development of postpartum depression [2].

Postpartum depression affects between 0.5% and 63.3% of mothers worldwide [3]. In Western countries, the prevalence varies from 10% to 15% in the first year after birth [4]. On the other hand, the overall pooled estimate of several studies conducted in the Middle East showed a much higher prevalence of 27% [5]. In Saudi Arabia alone, 32.8% of mothers experience postpartum depression [6]. Such high percentages pose significant health risks not only for mothers but also for their children [7,8].

Being frontline caregivers, maternity nurses are pivotal in identifying and preventing PPD. However, several studies have highlighted a deficiency in their knowledge and training, while others addressed challenges such as time constraints and early discharge protocols that make it difficult to recognize PPD in mothers following pregnancy. Therefore, educational interventions targeting maternity nurses' knowledge and attitudes toward PPD have been proposed as an effective strategy to ensure better identification and management of PPD and improve overall maternal and child health outcomes [7,9]. Such interventions involve various methods, including workshops, seminars, online courses, and simulation-based training [9]. To support the latter, a study by Zaki *et al*. [10] evaluated the effect of an educational program on the knowledge and attitudes of maternity nurses toward postpartum depression in Egypt. The program consisted of a 2-hour educational session on PPD, focusing on its symptoms, risk factors, and management strategies. The study found that the educational intervention significantly improved the knowledge and attitudes of maternity nurses toward postpartum depression. Another study by Almutairi *et al*. found that maternity nurses in Saudi Arabia lack the necessary knowledge and skills to identify and manage postpartum depression effectively [7,11]. This deficiency frequently leads to the underdiagnosis, or complete non-diagnosis, of many affected mothers in the Kingdom of Saudi Arabia [11].

Therefore, this study aimed to identify the specific educational needs of maternity nurses in Saudi Arabia while providing them with the necessary training to address postpartum depression effectively.

### Significance of the study

The significance of the current study lies in its potential impact on the quality of care provided to postpartum women. In Saudi Arabia, the prevalence of postpartum depression is significant, with various studies reporting rates as follows: 38.5% in Riyadh in 2014 [11], 20.9% in Jeddah [12], and 31.68% in Al-Madinah [13]. Maternity nurses play a crucial role in identifying and managing postpartum depression, as they are often the first point of contact for postpartum women in healthcare settings. Research has shown that maternity nurses may not be adequately prepared to address postpartum depression, as they may lack knowledge about its symptoms, risk factors, and management strategies [14,15]. Furthermore, negative attitudes and stigmatizing beliefs held by healthcare providers, including maternity nurses, toward mental illness can contribute to underreporting and undertreatment of postpartum depression [16,17]. Therefore, understanding the knowledge and attitudes of maternity nurses toward postpartum depression is important for improving the quality of care. This can help ensure that postpartum depression is identified and managed appropriately, leading to better maternal and child health outcomes.

### Aim of the study

This study aimed to investigate the impact of an educational intervention on improving the knowledge and attitude of mothers and maternity nurses on postpartum depression. More specifically, the study aimed to:

1. Assess the knowledge and attitudes of maternity nurses toward PPD pre/post-intervention.
2. Investigate the association between maternity nurses’ knowledge and their attitudes.
3. Compare the knowledge and attitudes of nurses pre- and post-intervention.
4. Examine the relationship between demographic and personal characteristics of nurses and their knowledge and attitudes toward postpartum depression.

### Research hypothesis

Hypothesis 1≠ Null Hypothesis (H0): There is no significant difference in the knowledge and attitudes of maternity nurses towards postpartum depression before and after the educational intervention.

Alternative Hypothesis (H1) ≠ There is a significant difference in the knowledge and attitudes of maternity nurses towards postpartum depression before and after the educational intervention.

Hypothesis 2: ≠ Null Hypothesis (H0): There is no significant relationship between demographic and personal characteristics of nurses (such as age, years of experience, education level, etc.) and their knowledge and attitudes about postpartum depression.

Alternative Hypothesis ≠ (H1): There is a significant relationship between demographic and personal characteristics of nurses (such as age, years of experience, education level, etc.) and their knowledge and attitudes about postpartum depression.

## MATERIAL AND METHODS

### Study design

This study utilized a quasi-experimental design with a one-group pre-post assessment approach. Since researchers can actively ensure that the difference between the pre- post-assessment of the participants is equivalent, this design was considered appropriate for establishing the cause-effect relationships between the independent (training program) and dependent variables (knowledge and attitudes of the maternity nurses).

### Setting and participants

A convenience sampling technique was employed to recruit 120 participating nurses who were available during the data collection period. The research was conducted at the Obstetric Ward 1 and the Obstetrics and Gynecology Inpatient Department at King Abdul-Aziz Medical City, Jeddah. Data were gathered from over 120 maternity unit and clinic employees, including 46 in Ward A, 46 in Ward B, and 28 in the clinic. Each inpatient ward accommodates 30 beds.

### Sample size calculation

The Rao soft sample size calculation was used to recruit 120 total staff nurses at in-patient maternity care units (*n* = 150). However, the minimum recommended sample size of nurses was 109 using the Rao soft sample size calculator (Sample Size Calculator by Rao soft, Inc.) with the following parameters: population size of 150, margin error of 5, confidence interval of 95%, and significance level of 0.05.

### Instruments used

To achieve the research objectives, three primary instruments were employed:

1. **Socio-demographic data:** This included age, educational level, occupational status, spouse education, job, household economic status, and sources for seeking help and information regarding postpartum depression.
2. **Postpartum Depression Literacy Scale (PoDLiS):** The Postpartum Depression Literacy Scale (PoDLiS) was developed by Mirsalimi *et al*. [18] to measure the knowledge of maternity nurses concerning postpartum depression. It consists of 31 statements divided into seven categories, with six statements measuring participants' ability to recognize postpartum depression, five statements assessing knowledge of risk factors and causes, another five items measuring knowledge and beliefs of self-care activities, four items measuring knowledge about professional help available and beliefs about professional help available, six items measuring participants' attitudes that facilitate recognition of postpartum depression and appropriate help, and five items measuring respondents' ability to seek information related to postpartum depression. According to the main developer, the psychometric analysis of the scale suggests that PoDLiS is a reliable and valid instrument for measuring postpartum depression literacy and can be used in studies of mental health literacy in women. Responses were measured on a 5-point Likert scale ranging from '5' (strongly agree) to '1' (strongly disagree), with higher scores indicating greater knowledge about PPD.
3. **Attitude assessment questionnaire:** Adapted from Kang *et al*.'s [19] study, the questionnaire featured 17 categories with five possible responses per category. However, during scoring, responses were condensed into three categories: disagree (combining strongly disagree and disagree), neutral, and agree (combining agree and strongly agree) [20]. Confirmatory factor analysis supported the scale's construct validity. Consequently, a total score was computed by averaging all items, with higher scores indicating more negative attitudes toward PPD.

Regarding validity and reliability, the study tools were initially tested for content validity and reliability by their primary developers, Mirsalimi *et al*. [18] and Alsabi *et al*. [20]. However, for the current study, the tools were utilized in English and subjected to review by a panel of experts to ensure accuracy and mitigate potential threats to the study's validity.

### Data collection procedure

Following official approval from the College of Nursing, Jeddah (CONJ), King Abdulaziz Medical Research Center (KAIMRC), and the International Research Board (IRB), data collection began. All nurses in inpatient maternity wards at the selected facilities were invited to join the study through leaflets and brief discussions. The study involved grouping nurses for pre/post-assessment and intervention, with 120 out of 150 nurses participating in the educational program. The participants were divided into six subgroups to accommodate their schedules and facilitate discussions. Each nurse was assigned a unique code for pre- and post-assessment, and the researcher facilitated both training sessions.

To control for bias, the following steps were employed:

- The initial assessment was completed using the fulfilled questionnaire.
- The informed consent, IRB approval, and participant codes were reviewed, and all participants were instructed to bring their code to the training to ensure that their first and second assessments could be compiled for data analysis.

### Program implementation

The intervention was implemented in several phases:

1. **Planning phase:** This phase involved the initial stages of developing the program, which included identifying the needs and goals of the program and determining the resources and timelines for implementation.
2. **Development phase:** This phase involved designing the program, including the program structure, the program content and materials, and selecting the appropriate delivery method.
3. **Implementation phase:** This phase involved the full implementation of the program, including delivering the program to the target audience, monitoring and evaluating the program's progress, and addressing any issues or challenges that arose. The program was overseen by the principal investigator (PI) and co-authors in collaboration with department supervisors, and it accommodated nurses' schedules. The training sessions were conducted virtually via Microsoft Teams and onsite in the maternity department for night shift nurses at 7:00 AM. Data collection took place from mid-October 2023 to the end of December 2023 for 12 sessions. Each group participated in 2-hour training sessions twice weekly, with the final 15 minutes dedicated to summarizing key learnings. Various training methods, including group discussions, modeling, role-playing, feedback, and real-life scenarios drawn from nurses' experiences, facilitated engagement and skill acquisition during the sessions. Pamphlets with resources were given to all attendees, one set in English for nurses and another in Arabic for other participants, to be shared with mothers at clinics or inpatient departments. The intervention program was split into two primary components:
  Firstly, there was a theoretical component that covered everything related to postpartum depression. This included a comprehensive overview of its causes, risk factors, signs and symptoms, complications and consequences, and management strategies. These strategies included both pharmacological and non-pharmacological options. Secondly, there was a practical component where maternity nurses were trained on how to use appropriate screening tools to identify women at risk of postpartum depression. They were also taught how to conduct a thorough assessment of the condition to establish a diagnosis and determine the severity of the condition. Effective communication skills were also emphasized so that nurses could properly identify and manage postpartum depression in a sensitive and non-judgmental manner. Maternity nurses were also trained on the importance of follow-up and referral of women with postpartum depression to appropriate healthcare providers such as psychiatrists, psychologists, or social workers. Additionally, self-care and self-management strategies were discussed to prevent burnout and ensure the well-being of nurses while caring for women with postpartum depression.
4. **Evaluation phase:** This phase assessed the effectiveness and impact of the program, including evaluating the program's outcomes, measuring participant satisfaction, and identifying areas for improvement.

### Data management and analysis plan

Data were structured and validated before analysis using SPSS software. Categorical variables were described using frequency and percentages, while continuous and scale variables were summarized with means and standard deviations (SD). Bar charts were generated to visually represent the variables. The analysis employed various statistical tests including independent *t*-tests, ANOVA, paired t-tests, and Cohen effect size calculation. Additionally, the percentage change in means between pre- and post-knowledge and attitude scales was computed. A significance threshold of *P* < 0.05 was applied to determine statistical significance.

## RESULTS

Table 1 presents a breakdown of the participants' demographic information. A considerable proportion (51.7%) were aged between 30 and 39 years, with 62 individuals falling into this age group. The majority of participants (64.2%) had a bachelor's degree, while 85.8% had an occupational status of SN1 (Staff Nurse 1). Regarding experience, 27.5% of individuals were in the field for 11-15 years. Malaysian nationals made up the largest nationality group, accounting for 35% of the participants. As for marital status, 65.8% of participants were married, and 52.5% had 1-3 children. Regarding household income, 51.7% of participants had a household income in the range of 5000-10000. Finally, most participants (84.2%) did not participate in workshops on PPD.

**Table 1:** Distribution of study participants according to their demographic characteristics (*n* = 120)

Variables	No (%)
**Age**	
20-29	16 (13.3)
30-39	62 (51.7)
40-49	19 (15.8)
50 and above	23 (19.2)
**Educational level**	
Diploma	43 (35.8)
Bachelor and above	77 (64.2)
**Occupational status**	
SN1	103 (85.8)
SN2	17 (14.2)
**Years of experience**	
0-5	19 (15.8)
6-10	30 (25.0)
11-15	33 (27.5)
16-20	12 (10.0)
21-25	15 (12.5)
26-30	11 (9.2)
**Nationality**	
Saudi	26 (21.7)
Malaysian	42 (35.0)
Filipino	39 (32.5)
Others	13 (10.8)
**Marital status**	
Married	79 (65.8)
Single	33 (27.5)
Divorced	6 (5.0)
Widowed	2 (1.7)
**No of children**	
0	14 (11.7)
1-3	63 (52.5)
4-6	10 (8.3)
NA	33 (27.5)
**Household income status**	
Less than 5000	15 (12.5)
5000-10000	62 (51.7)
11000-20000	36 (30.0)
More than 20000	7 (5.8)
**Attending a workshop on PPD**	
Yes	19 (15.8)
No	101 (84.2)

**Figure 1:**
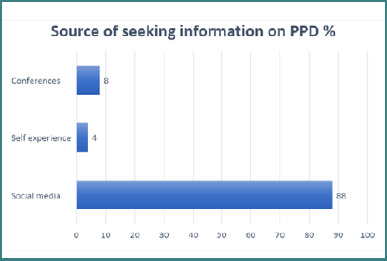
Preferred sources for information on PPD among study participants (*n* = 120)

The bar graph illustrates the sources people seek information on PPD (Figure 1). The graph shows that social media was the predominant source of information regarding PPD, with 88% of the respondents using it. This may reflect the popularity and accessibility of social media platforms, as well as the potential benefits of online support groups and peer interactions for women experiencing PPD.

**Figure 2:**
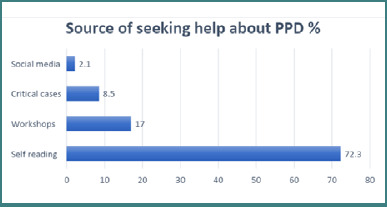
Primary methods for seeking help regarding PPD among study participants (n = 120)

The horizontal bar (Figure 2) displays the sources from which individuals seek help for PPD. Self-reading was the most common method, with 72.3% of the respondents using it. This may indicate that people prefer to learn about PPD on their own or that they have limited access to other sources of help.

Table 2 presents the changes in knowledge and attitudes towards postpartum depression before and after the intervention. The results demonstrate significant improvements in various areas among the participants. For instance, the recognition of PPD increased by 8.02%, while there was a 10.09% increase in understanding risk factors and causes and a 6.59% enhancement in knowledge about self-care activities. Moreover, participants showed a 7.03% improvement in seeking professional help for PPD and a 9.76% increase in recognition facilitation. The data also revealed a 5.47% increase in seeking information about PPD, indicating proactive engagement. Overall, there was a 5.75% increase in participants' knowledge about PPD, with attitude changes being the most significant, as demonstrated by a substantial 15.43% increase post-intervention. These results suggest that the intervention effectively improved knowledge and attitudes towards PPD, which is supported by statistically significant *P* values (ranging from 0.001 to 0.002) and moderate to large effect sizes (ranging from -0.37 to -0.68).

**Table 2:** Pre- and post-intervention comparison of knowledge and attitudes regarding PPD among participants (n = 120)

Item	Pre-intervention	Post-intervention	% of Change	*P* value	Effect size
Recognition of PPD	3.49 ± 0.33	3.77 ± 0.43	8.02	0.001	-0.52
Risk factors and causes	3.47 ± 0.36	3.82 ± 0.33	10.09	0.001	-0.68
Self-care activities	3.34 ± 0.43	3.56 ± 0.33	6.59	0.001	-0.43
Professional help	3.13 ± 0.52	3.35 ± 0.46	7.03	0.001	-0.37
Recognition facilitation	2.46 ± 0.67	2.70 ± 0.59	9.76	0.002	-0.43
Seeking information	3.29 ± 0.44	3.47 ± 0.41	5.47	0.001	-0.44
Overall knowledge of PPD	2.61 ± 0.24	2.76 ± 0.33	5.75	0.001	-0.54
Attitude towards PPD	1.88 ± 0.37	2.17 ± 0.48	15.43	0.001	-0.53

**Figure 3:**
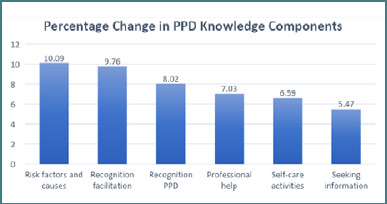
Changes in knowledge scale following the educational intervention (*n* = 120)

Figure 3 presents the percentage change in various domains of postpartum depression awareness among maternity nurses. The most notable improvement was understanding the risk factors and causes of PPD, with a 10.09% increase. This was followed by a 9.76% increase in the ability to facilitate recognition of PPD symptoms and seek appropriate assistance. On the other hand, seeking reliable sources of information about PPD showed the least progress, with a 5.47% increase.

Table 3 presents the correlation between pre-and post-intervention knowledge and attitudes toward PPD. The results show that the intervention positively affected the knowledge of risk factors and causes of PPD, as indicated by a significant increase in the correlation coefficient from 0.090 to 0.244 (*P* < 0.05). However, the intervention did not significantly impact the recognition, self-care, professional help, and seeking information domains, as the correlation coefficients either decreased or remained low after the intervention. Additionally, the overall PPD knowledge had a positive but reduced relationship with attitude post-intervention (*r* = 0.147).

**Table 3:** Correlation between PPD knowledge and attitude among study participants (*n* = 120)

Item	Pre-intervention	Post-intervention
Recognition PPD	0.100	0.057
Risk factors and causes	0.090	0.244*
Self-care activities	0.149	0.048
Professional help	0.345*	0.037
Recognition facilitation	0.393*	0.295*
Seeking information	0.207*	0.040
Overall knowledge of PPD	0.324*	0.147

* Significant correlation

Table 4 presents the average change in PPD knowledge scores based on participants' basic characteristics. Nationality was the only factor with significant variance, with Filipino participants displaying the highest mean change (0.29) and Saudi participants exhibiting the lowest mean change (0.05). Conversely, the results demonstrate no considerable difference in PPD knowledge associated with age, educational level, occupational status, years of experience, marital status, number of children, household income status, or attendance at PPD workshops.

**Table 4:** Correlation between overall PPD knowledge changes and basic participant characteristics (*n* = 120)

Variable	Mean change	*P* value
**Age**		0.178
20-29	0.02	
30-39	0.14	
40-49	0.19	
50 and above	0.27	
**Educational level**		0.217
Diploma	0.10	
Bachelor	0.19	
**Occupational status**		0.596
SN1	0.17	
SN2	0.11	
**Years of experience**		0.190
0-5	0.08	
6-10	0.22	
11-15	0.08	
16-20	0.27	
21-25	0.06	
26-30	0.31	
**Nationality**		0.025
Saudi	0.05	
Malaysian	0.09	
Filipino	0.29	
Others	0.21	
**Marital status**		0.821
Married	0.15	
Not married	0.17	
**No of children**		0.770
0	0.17	
1-3	0.13	
4-6	0.22	
**Household income status**		0.516
Less than 5000	0.21	
5000-10000	0.17	
11000-20000	0.09	
More than 20000	0.29	
**Attending a workshop on PPD**		0.269
Yes	0.24	
No	0.14	

According to Table 5, participants' basic characteristics influenced the overall attitude changes towards PPD. The intervention positively affected attitudes, particularly within social support networks, as indicated by the overall mean change. However, attending a workshop on PPD was the only variable that significantly affected the mean change in attitude. Interestingly, those who did not attend a workshop on PPD had a greater attitude improvement than those who did attend. Demographic backgrounds such as age, educational level, occupational status, years of experience, nationality, marital status, number of children, or household income status did not significantly impact attitude changes.

**Table 5:** Correlation between overall PPD attitude changes and basic participant characteristics (*n* = 120)

Variable	Mean change	*P* value
**Age**		0.316
20-29	0.27	
30-39	0.39	
40-49	0.10	
50 and above	0.25	
**Educational level**		0.886
Diploma	0.30	
Bachelor	0.28	
**Occupational status**		0.757
SN1	0.29	
SN2	0.25	
**Years of experience**		0.845
0-5	0.23	
6-10	0.41	
11-15	0.26	
16-20	0.25	
21-25	0.21	
26-30	0.30	
**Nationality**		0.671
Saudi	0.26	
Malaysian	0.37	
Filipino	0.25	
Others	0.19	
**Marital status**		0.918
Married	0.28	
Non-married	0.29	
**No of children**		0.445
0	0.31	
1-3	0.30	
4-6	0.05	
**Household income status**		0.537
Less than 5000	0.29	
5000-10000	0.31	
11000-20000	0.31	
More than 20000	-0.08	
**Attending a workshop on PPD**		0.026
Yes	0.03	
No	0.34	

## DISCUSSION

The study evaluated the effectiveness of an educational program in enhancing the knowledge and attitudes of maternity nurses regarding PPD. The intervention, comprising interactive lectures, videos, role-playing, and case studies, addressed misconceptions and stigma associated with PPD.

Results showed a significant improvement in knowledge and attitudes among the participants, indicating a positive change in beliefs and perceptions about PPD. These findings are consistent with previous research highlighting the effectiveness of educational interventions in improving PPD-related knowledge and attitudes among maternity nurses.

The study also found an increase in understanding of proper PPD screening among prenatal nursing personnel following the intervention, which aligns with the work of Jewell *et al*. [21]. Similarly, the study supports the findings of Reid *et al*. [22], who highlighted the benefits of educational programs focused on PPD screening tools for healthcare professionals working with new mothers.

It is important to approach the findings with caution as there are limitations and inconsistencies in the literature. Future research should include randomized controlled trials with diverse samples and broader scopes to make the results more reliable and applicable. Longitudinal studies with multiple measurements are also necessary to evaluate the intervention's long-term effects and how it affects participants' professional practices. A meta-analysis of existing studies could also provide better insight into best practices for designing and implementing educational interventions that target PPD improvement knowledge and attitudes among maternity nurses.

Regarding the intervention's effectiveness, Table 2 shows significant improvements in participants' knowledge and attitudes toward PPD, which aligns with Bena *et al*.'s [23] findings on the relationship between perceived competence, knowledge, attitudes, training, and the ability to manage PPD. However, Table 3 indicates discrepancies in participants' understanding of specific aspects of PPD, such as self-care and symptom detection.

Table 1 shows that Malaysians make up over one-third of the study population (35%), which highlights the challenges faced by the Saudi nursing profession, such as a shortage of native nurses [24,25]. This shortage leads to reliance on foreign healthcare professionals, creating communication difficulties. Pizzuti *et al*. [26] emphasized the importance of social media as a source of PPD information, which presents an opportunity for healthcare education. Figure 2 depicts the common sources individuals turn to for help with PPD, which is consistent with Manso-Córdoba *et al*.'s [27] findings on women concealing their suffering due to the expectation-experience gap.

Figure 3 analyzes changes in PPD knowledge among maternity nurses, which suggests significant improvements in understanding risk factors and causes. This finding aligns with Bridget *et al*. [28]. Notably, nationality emerged as a significant variable, with Filipinos exhibiting the most significant average change. O'Brien *et al*. [29] advocate for the early integration of cultural aspects into nursing education, highlighting the importance of cross-cultural interactions during clinical placements.

Moreover, Table 5 emphasizes the impact of educational programs on participants' attitudes toward PPD. Attendance at PPD workshops is pivotal in attitude change, as per Phoosuwan *et al*. [30]. The findings of this study support previous research on the influence of participant characteristics on improvements in PPD knowledge [31-34]. Gomez *et al*. [35] noted nationality as a significant factor in knowledge level changes, while Chow *et al*. [36] and Duan *et al*. [37] highlighted demographic variables such as age, education, and experience as influential factors.

Structured educational programs have been shown to enhance attitudes toward PPD, as evidenced by Jackson *et al*. [38] and Brown *et al*. [39]. However, studies by Miller *et al*. [40] and Garcia *et al*. [41] reported mixed results, suggesting that individual beliefs and prior exposure to PPD workshops may also play a role.

This study emphasizes the importance of continuous training and skill development in building the confidence and competence of healthcare professionals. This finding is consistent with the research of Fernandez *et al*. [42] and Carter *et al*. [43]. However, sustaining changes in self-efficacy may present challenges, as Johnson *et al*. [44] and Green *et al*. [45] have noted. They recommend ongoing support and reinforcement strategies.

### Limitations

Although the study provided valuable insights into the level of knowledge and attitudes of maternity nurses, it did not have a control group or a randomized design, which could have reduced the impact of other variables. Therefore, future research should incorporate a control group or a randomized design to strengthen the ability to establish cause and effect in the intervention. Additionally, it is crucial to examine the long-term effects of educational intervention on nurses' knowledge, attitudes, and behaviors related to PPD care and support. By investigating sustained impacts over time, we can better understand the effectiveness of the intervention in promoting lasting changes in nursing practices and ultimately improving outcomes for mothers with PPD.

### Recommendations

It is crucial to emphasize the importance of ongoing education and training programs focused on PPD for maternity nurses. Tailored workshops and seminars should be designed to address the needs and challenges maternity nurses face in recognizing and addressing PPD. Additionally, it is essential to encourage maternity nurses to seek information on PPD from diverse sources beyond social media, such as evidence-based literature, professional organizations, and accredited training modules. Emphasizing the importance of critical appraisal and validation of information obtained from online platforms is vital in ensuring the accuracy and reliability of the information. Moreover, promoting interdisciplinary collaboration between maternity nurses, mental health professionals, obstetricians, pediatricians, and community support services is essential in facilitating early detection, intervention, and holistic management of PPD. This interdisciplinary approach can significantly improve the overall outcomes for mothers experiencing PPD.

### Nursing implications

*Enhanced patient care:* Improved knowledge and attitudes among maternity nurses regarding PPD can lead to enhanced patient care outcomes, including early identification of at-risk individuals, timely intervention, and provision of appropriate support and resources to women experiencing PPD.

*Reduced stigma and barriers to care:* By fostering a supportive and empathetic care environment, maternity nurses can help reduce the stigma associated with PPD and encourage women to seek help and support without fear of judgment or discrimination.

*Empowerment of women and families:* Equipping maternity nurses with the necessary knowledge and skills to address PPD can empower women and their families to navigate the challenges associated with perinatal mental health and access the resources and support they need to promote maternal well-being and infant health.

*Advocacy and policy development:* Maternity nurses play a vital role in advocating for policy changes and resource allocation to support the implementation of evidence-based practices and interventions for preventing, detecting, and managing PPD at both institutional and community levels.

*Active advocacy:* By actively engaging in advocacy efforts, maternity nurses can help create supportive environments, such as specialized wards for mothers affected by PPD.

## CONCLUSION

The study showed that the educational program significantly improved the knowledge and attitudes of maternity nurses toward PPD. The impact was most noticeable in the nurses' attitudes, indicating a positive shift in their perceptions of PPD. The study emphasized the connection between knowledge and attitudes, influenced by factors such as nationality and previous attendance at PPD workshops. The findings highlight the importance of targeted educational efforts to equip maternity nurses with the necessary skills and awareness to identify, assess, and support women with PPD. This underscores the significance of ongoing professional development in maternal healthcare.

## Data Availability

Further data is available from the corresponding author upon reasonable request.
